# Comparative Analysis of the Soluble Proteome and the Cytolytic Activity of Unbleached and Bleached *Millepora complanata* (“Fire Coral”) from the Mexican Caribbean

**DOI:** 10.3390/md17070393

**Published:** 2019-07-03

**Authors:** Víctor Hugo Hernández-Elizárraga, Norma Olguín-López, Rosalina Hernández-Matehuala, Andrea Ocharán-Mercado, Andrés Cruz-Hernández, Ramón Gerardo Guevara-González, Juan Caballero-Pérez, César Ibarra-Alvarado, Judith Sánchez-Rodríguez, Alejandra Rojas-Molina

**Affiliations:** 1 Posgrado en Ciencias Químico Biológicas, Facultad de Química, Universidad Autónoma de Querétaro, Cerro de las Campanas S/N, C.P. 76010 Querétaro, Querétaro, México; 2 Laboratorio de Investigación Química y Farmacológica de Productos Naturales, Facultad de Química, Universidad Autónoma de Querétaro, Cerro de las Campanas S/N, C.P. 76010 Querétaro, Querétaro, México; 3 Laboratorio de Biología Molecular. Escuela de Agronomía, Universidad de La Salle Bajío, Av. Universidad 15 602, Colonia Lomas del Campestre, C.P. 37150 León, Guanajuato, México; 4 C.A Ingeniería de Biosistemas, Facultad de Ingeniería-Campus Amazcala, Universidad Autónoma de Querétaro, Carr. Chichimequillas-Amazcala Km. 1, S/N, C.P. 76265 Amazcala, El Marqués, Querétaro, México; 5 Facultad de Química, Universidad Autónoma de Querétaro, Cerro de las Campanas S/N, C.P. 76010 Querétaro, Querétaro, México; 6 Unidad Académica de Sistemas Arrecifales Puerto Morelos, Instituto de Ciencias del Mar y Limnología, Universidad Nacional Autónoma de México, Prolongación Niños Héroes S/N, Puerto Morelos, C.P. 77580 Quintana Roo, México

**Keywords:** proteomics, toxins, coral bleaching, hydrozoa, *Millepora complanata*

## Abstract

Coral bleaching caused by global warming has resulted in massive damage to coral reefs worldwide. Studies addressing the consequences of elevated temperature have focused on organisms of the class Anthozoa, and up to now, there is little information regarding the mechanisms by which reef forming Hydrozoans face thermal stress. In this study, we carried out a comparative analysis of the soluble proteome and the cytolytic activity of unbleached and bleached *Millepora complanata* (“fire coral”) that inhabited reef colonies exposed to the 2015–2016 El Niño-Southern Oscillation in the Mexican Caribbean. A differential proteomic response involving proteins implicated in key cellular processes, such as glycolysis, DNA repair, stress response, calcium homeostasis, exocytosis, and cytoskeleton organization was found in bleached hydrocorals. Four of the proteins, whose levels increased in bleached specimens, displayed sequence similarity to a phospholipase A2, an astacin-like metalloprotease, and two pore forming toxins. However, a protein, which displayed sequence similarity to a calcium-independent phospholipase A2, showed lower levels in bleached cnidarians. Accordingly, the hemolytic effect of the soluble proteome of bleached hydrocorals was significantly higher, whereas the phospholipase A2 activity was significantly reduced. Our results suggest that bleached *M. complanata* is capable of increasing its toxins production in order to balance the lack of nutrients supplied by its symbionts.

## 1. Introduction

Coral reefs are megadiverse ecosystems that offer a great variety of services to the human population surrounding them [1]. Calcareous structures formed by corals provide livelihood to a large range of marine species [2]. The hydrozoan *Millepora complanata* is an important reef-forming organism that is widely distributed in the Caribbean Sea. This organism belongs to the group commonly known as “fire corals”, which when getting into contact with humans are capable of producing severe burns, blisters, and pain [3]. Cnidarians are widely recognized as an important source of structurally diverse metabolites, which might represent novel leads for the development of new drugs and biotechnological tools. One of the most remarkable features of cnidarians is their ability to synthesize cnidocystic and non-cnidocystic toxins (neurotoxins, enzymes, and pore-forming toxins) that induce toxic and immunological reactions [4,5]. Most of these toxins are contained within the nematocysts, and are implicated in both cnidarian defense and prey capture [6]. Previous studies carried out by our research group demonstrated that *M. complanata* produces hemolysins, phospholipases A2 (PLA2), and proteases [7,8].

Coral reef forming cnidarians live in mutualistic symbiosis with photosynthetic algae of the genus *Symbiodinium*, commonly named zooxanthellae. In this symbiotic relationship, algae provide approximately 95% of nutrients or metabolic requirements (by photosynthetically fixed carbon) to their cnidarian host [9,10,11,12]. Environmental stressors, such ocean acidification, elevated salinity, UV radiation, and high temperature can lead to the breakdown of the coral-algae symbiosis. This phenomenon, commonly known as “coral bleaching” [13,14,15,16], results from the loss of photosynthetic symbionts or algae pigments from cnidarian host cells [17]. It has been well documented that worldwide coral bleaching events are among the most deleterious effects of global warming, putting the survival of coral reef at serious risk [18,19,20,21]. In the last 100 years, the average temperature on earth has increased by about 1 °C, and according to records of the US National Oceanic and Atmospheric Administration (NOOA), 2015–2016 were the warmest years recorded in the Earth’s history. Particularly, during the El Niño-Southern Oscillation (ENSO), severe coral bleaching events have occurred due to seawater temperature rise [22].

Since the first studies about the consequences of thermal stress were carried out, it has been widely demonstrated that after a bleaching event, different cnidarian cellular processes are affected [2,9,13,14,15,16,17,18,19,20,23]. Thermal stress induced upregulated expression levels of antioxidant enzymes (e.g., ascorbate peroxidase, catalase, superoxide dismutase) and heat shock proteins (e.g., HSP70), which are directly correlated with molecular mechanisms responsible for repairing cellular and tissue damage [24]. Stress induced by high UV radiation and elevated temperatures in *Montastraea faveolata* caused host DNA damage correlated to p53 gene expression, as well as decreased concentration of D1 protein and photosynthetic pigments in the algal symbionts [25]. In addition, *M. faveolata* exposed to high solar radiation showed diminished concentration of mycosporine-like amino acids, whose origin, whether from cnidarians or from their symbionts, was not determined [25]. It has also been proven that thermal stress and UV-light cause lower enzymatic activity of ribulose-1,5-bisphosphate carboxylase oxygenase (Rubisco) [26] and injury to the thylakoidal membranes by causing oxidative stress in *Symbiodinium* cells [27].

Several genomic and transcriptomic studies conducted in Anthozoa species have shown that thermal stress modifies the expression of genes and transcripts related to growth arrest, chaperone activity, nucleic acid stabilization, removal of damaged macromolecules, metabolism, antioxidant mechanisms, and immune system in both hosts and symbionts [28,29,30,31,32,33,34,35,36,37]. On the other hand, although there has only been a few proteomic studies on the consequences of elevated temperature on corals, these have provided evidence that important changes occur in the expression of proteins of reef-forming Anthozoans during bleaching [38,39,40,41]. In *Acropora palmata* and *Acropora microphthalma* the proteins, which showed differential expression after bleaching, participate in important cellular processes and components, which include: stress response, UV response, amino acid synthesis, transcription factors, immunity, apoptosis, biomineralization, cytoskeletal, cell cycle, oxidative phosphorylation, anti-oxidant proteins, endo-exo phagocytosis, and calcification [39,40]. Another study indicated that when *Pocillopora acuta* was subjected to experimental thermal stress, several proteins involved in cytoskeletal structure, immunity, and metabolism were differentially expressed [41]. It has been observed that heat stress causes damage to the coral host tissue, compromising the physiologic integrity of epithelium in *Acropora hyacinthus* [42]. Changes caused by thermal stress have been observed in the proteome of organisms from other phyla, such as the benthic foraminifera *Amphistegina gibbosa* [43]. Not only the transcriptome and the proteome, but also the metabolome of both partners of the symbiosis is affected by thermal stress. Significant differences have been observed after bleaching in the lipid (e.g., cell structural lipids) and metabolite profiles (e.g., carbohydrates and signaling compounds) in *Pocillopora damicornis* [44] and *Acropora aspera*, such metabolites are involved in biochemical reactions related to molecular regulation during exposure to environmental stress in cnidarians [45,46]. A metabolomics analysis of the symbiotic anemone *Aiptasia* sp. confirmed the results obtained from the study of reef forming cnidarians, indicating that thermal stress significantly alters central metabolism, oxidative state, and cell structure [47].

Studies aimed at evaluating the influence of elevated temperature on the cellular processes of reef forming cnidarians have focused on Anthozoa species, and up to now, very little is known about the cellular response of Hydrozoa species to thermal stress. In a previous study carried out by our research group, we analyzed the impact of thermal stress on the soluble proteomic profile and cytolytic activity of *Millepora alcicornis*. We found that the levels of 17 key proteins, tentatively identified as related to exocytosis, calcium homeostasis, cytoskeletal organization were modified in bleached *M. alcicornis*. Moreover, the protein levels of potential toxins, including a metalloprotease, a phospholipase A2 (PLA2), and an actitoxin were also altered [48]. It is obviously very important to continue studying the consequences of high water temperature on hydrocorals. In this context, the present study was undertaken to investigate the effect of the 2015–2016 El Niño-Southern Oscillation on the soluble proteomic profile and cytolytic activity of *Millepora complanata* from the Mexican Caribbean through a proteomic approach, in order to contribute to the broader understanding of the molecular processes involved in the response of reef-forming organisms of the class Hydrozoa to global warming.

## 2. Results

In order to determine changes in the soluble proteomic profile and cytolytic activity of *M. complanata* that experienced bleaching during the 2015–2016 El Niño-Southern Oscillation, the method chosen for the extraction of hydrocoral’s proteins involved osmotic shock in bidistilled water, which causes the discharge of the nematocysts content. The soluble proteomes from unbleached and bleached specimens obtained in this way were examined by using high-resolution two-dimensional electrophoresis (2DE). Protein spots with different abundance were subjected to MALDI-TOF/TOF mass spectrometry analysis. In addition, to explore if the cytolytic activity produced by the soluble proteome of this hydrocoral was affected, the hemolytic and the PLA2 activities of the proteomes obtained from unbleached and bleached *M. complanata* specimens were assessed. 

### 2.1. Sample Collection and Soluble Proteome Extraction

**Figure 1:**
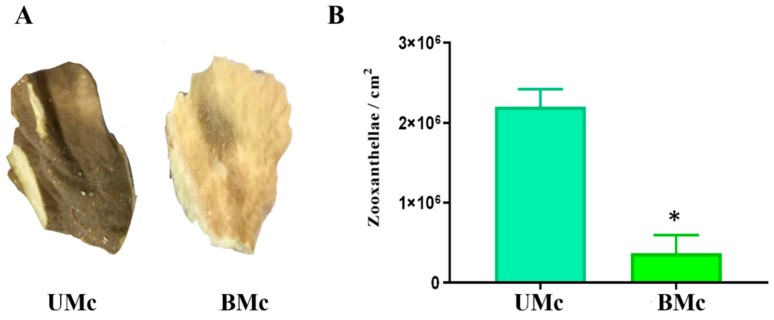
(**A**) Photographs of representative fragments of unbleached (UMc) and bleached (BMc) *M. complanata* collected in 2016 in the Mexican Caribbean. (**B**) Symbiont density quantified (*n* = 3) and expressed per cm^2^ for UMc and BMc. Data are mean ± SEM. (*) Indicate significant difference (*p* < 0.05) in symbiont density between UMc and BMc. Photographs from UMc and BMc specimens were taken by Víctor Hugo Hernández-Elizárraga and Norma Olguín-Lopez.

Representative bleached (BMc) and unbleached (UMc) samples of M. complanata colonies are shown in Figure 1A. All samples were cut from the edges of plate-like colonies. Protein contents of the soluble proteomes from UMc and BMc were 31.04 ± 1.30 µg of protein/mg lyophilized and 22.02 ± 0.70 µg of protein/mg lyophilized, respectively.

### 2.2. Determination of the Degree of Bleaching

Results derived from symbiont quantification are shown in Figure 1B. In the case of unbleached specimens, the average number of symbionts per square centimenter was 2.2 ± 0.12 × 10^6^, while the average number for bleached hydrocorals was 3.6 ± 1.3 × 10^5^. Bleached M. complanata showed a statistically significant decrease (*p* < 0.05) in zooxanthellae population compared to unbleached organisms.

### 2.3. Electrophoresis SDS-PAGE

**Figure 2:**
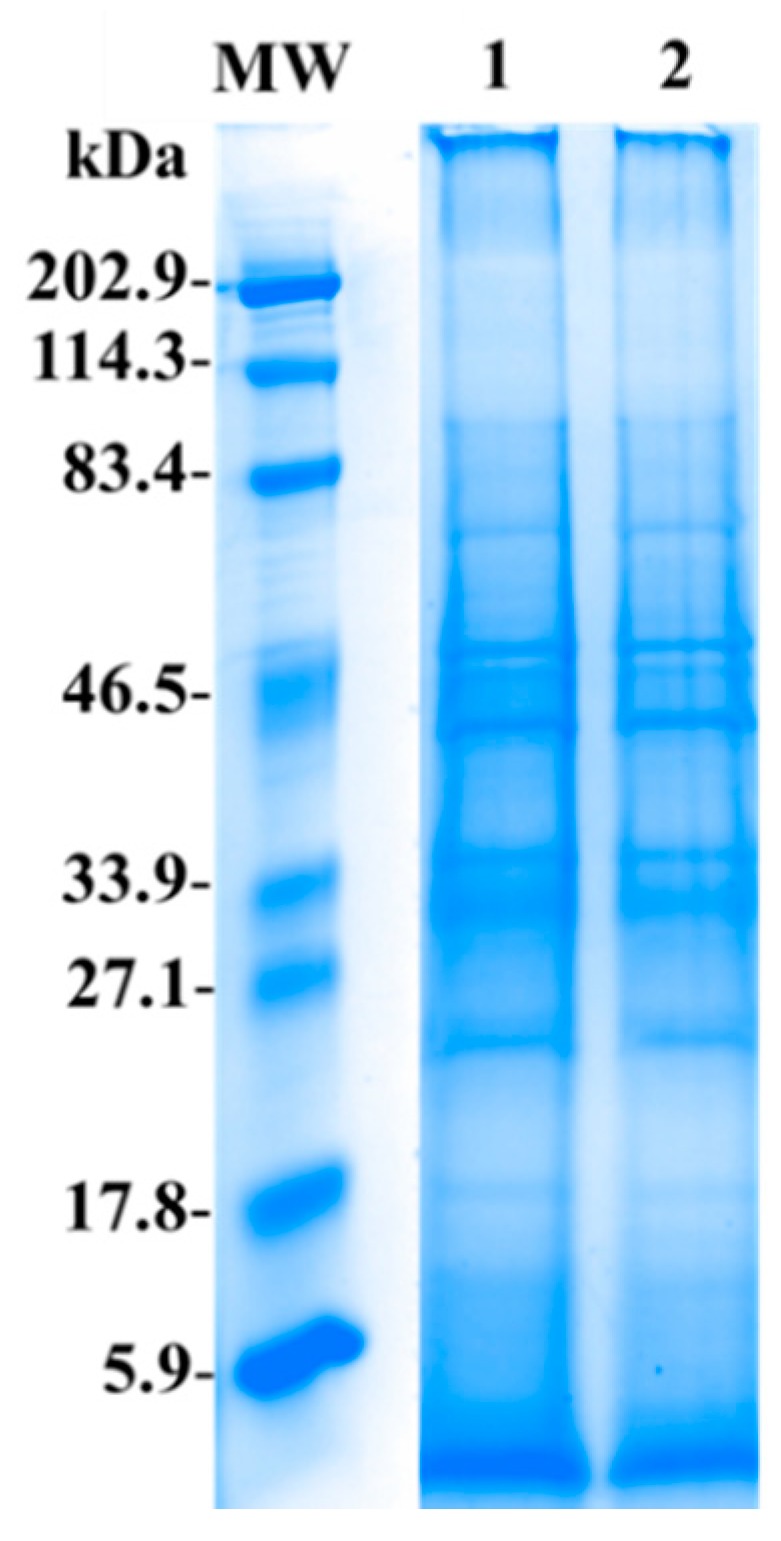
Representative electrophoresis gel showing the protein profiles of the soluble proteomes from unbleached [UMc (1)] and bleached [BMc (2)] M. complanata specimens. Samples (80 μg of protein) were separated by one dimensional SDS-PAGE using 12% (w/v) polyacrylamide under non-reducing conditions. (MW) Molecular weight marker. Protein bands were stained using Coomassie Blue R-250. MW and samples 1 and 2 were resolved in the same gel, lanes with lower protein concentrations were removed.

Soluble proteome profiles from unbleached and bleached M. complanata specimens under denaturing and reducing conditions are shown in Figure 2. Both soluble proteomes contained proteins with a broad range of molecular weights (5.9–202.9 kDa). Prominent protein bands ranged in size between 20 and 80 kDa.

### 2.4. Two-Dimensional High-Resolution Gel Electrophoresis (2DE-PAGE)

**Figure 3:**
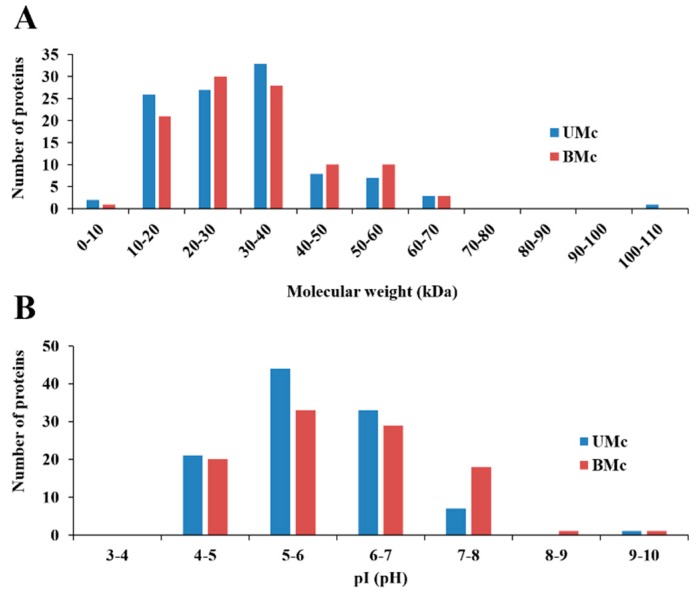
Isolectric point (pI) and molecular weight distribution of proteins found in the soluble proteomes from unbleached (UMc) (*n* = 3) and bleached (*n* = 3) specimens (BMc) of *M. complanata*. (**A**) Distribution graph of molecular weights. (**B**) Distribution graph of isoelectric points.

**Figure 4:**
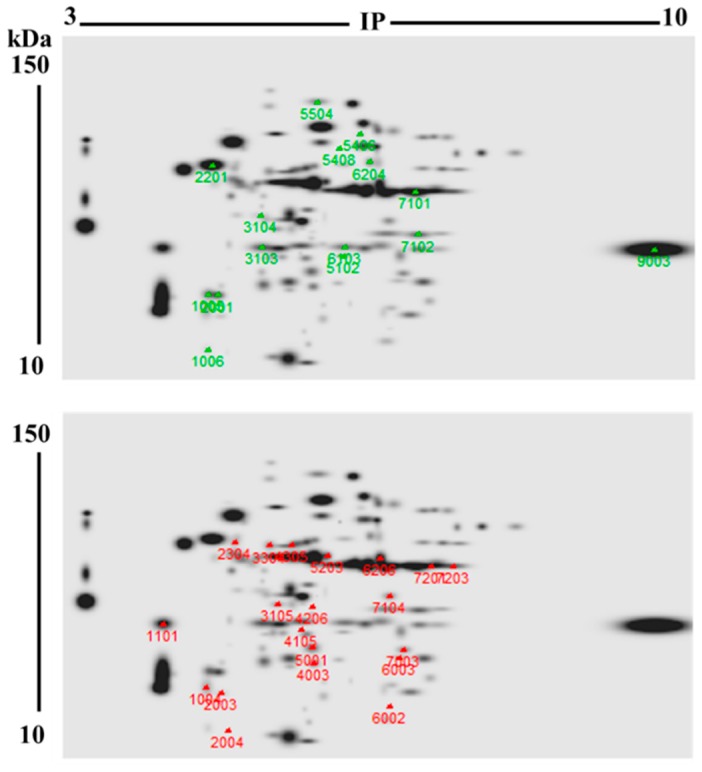
Representative 2DE “master” gels of M. complanata-soluble proteomes. This master gel displays combined proteomes from both bleached (*n* = 3) and unbleached conditions (*n* = 3). The marks and numbers on the 2DE gels show the differentially expressed proteins (fold change ≥ 2). Significant changes in the levels of 35 proteins were observed. Protein spots in *M. complanata*-soluble proteome which exhibited higher levels in bleached samples are indicated as green marks. Protein spots whose levels were lower in bleached hydrocorals are indicated as red marks.

DE-PAGE analysis evidenced that proteomes from unbleached (103 protein spots) and bleached (95 proteins spots) hydrocorals showed different protein profiles. Most of the proteins had isoelectric points (pI) and molecular weight values ranging from 4–8 and 10–40 kDa, respectively (Figure 3). 71 protein spots matched in both soluble proteomes, while 35 were differentially expressed (fold change > 2) in bleached specimens, fifteen of these proteins were up-regulated, while twenty were down-regulated (Figure 4).

### 2.5. Identification of Proteins Whose Levels Changed in Bleached M. complanata

Our results demonstrated that levels of thirty-five proteins were modified in bleached M. complanata. Fifteen differentially abundant protein spots were analyzed by MALDI-TOF/TOF, the ProteinPilot software, and BLASTp (Table 1). The other protein spots were not examined due to their low concentration. Identified proteins were classified, according to their function, into 8 groups: toxins, primary metabolism, DNA repair, cytoskeleton components, signaling proteins, stress response, redox homeostasis, and exocytosis proteins. Bleached hydrocorals exhibited higher levels of alfa enolase, UV DNA endonuclease, HSP70, peroxiredoxina-6, and exocyst complex component 4 like protein, while the abundance of triosephosphate isomerase, DNA endonuclease repair XPF, actin, calmodulin, and hypothetical protein NEMVEDRAFT_v1g45829) was diminished. Interestingly, bleached M. complanata showed increased levels of 4 proteins, which possess amino acid sequences that resemble the primary structure of toxins such as a secreted acidic PLA2 PA4, echotoxin-2, DELTA-actitoxin-Oor1b, and astacin-like metalloprotease toxin 5. Additionally, abundance of a protein that bears sequence similarity with an acidic calcium-independent phospholipase A2-like protein was decreased in hydrocorals that underwent bleaching. 

### 2.6. Effect of Elevated Sea Temperature on the Cytolytic Activity of Unbleached- and Bleached M. complanata-Soluble Proteomes

**Figure 5:**
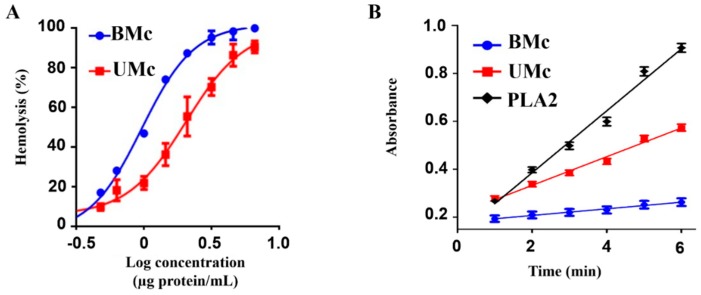
(**A**) Concentration-response curves showing the hemolytic activity elicited on rat erythrocytes by the soluble proteomes of unbleached (UMc) and bleached (BMc) specimens of *M. complanata*. (**B**) Progress curve for the PLA2 activity induced by UMC- and BMc- soluble proteomes at 414 nm. Positive control: PLA2 from Apis mellifera. Each point of the curves represents the mean of three replicates.

Both soluble proteomes from unbleached and bleached M. complanata induced a concentration-dependent hemolysis (Figure 5A). However, the concentration necessary to produce 50% hemolysis (HU50) of the soluble proteome from bleached M. complanata was significantly lower than that of the unbleached M. complanata-soluble proteome, indicating that bleached hydrocorals possessed higher hemolytic activity. In contrast, the PLA2 elicited by the soluble proteome of bleached M. complanata was significantly reduced (Figure 5B and Table 2).

## 3. Discussion

During the 2015–2016 El Niño-Southern Oscillation, the highest shallow sea water temperatures were recorded and a severe impact on climate and weather due to this event was documented [49,50,51]. The 2015–2016 ENSO brought weather conditions that triggered coral bleaching and mortality worldwide [51]. In particular, the Caribbean coral reef ecosystems experienced severe bleaching [21,52]. It is well known that reef-forming cnidarians have an ability to counteract the damaging effects of thermal stress varies between organisms from different families or between species. For example, it has been demonstrated that species of the families Acroporidae, Pectiniidae, Alcyonacea, Merulinidae, and Mussidae are particularly vulnerable to the deleterious effects of temperature stress [53]. While organisms such as the soft coral *Sarcophyton ehrenbergi*, the massive coral *Porites cylindrical*, and the blue coral *Heliopora coerulea* have shown greater resistance to high temperature [54,55,56]. Comprehensive reef surveys have revealed that *Millepora* species (class Hydrozoa) are very susceptible to the bleaching phenomenon [57,58,59]. Therefore, considering that thermal stress is often induced during episodic heating events and the great ecological importance of hydrocorals, the aim of the present study was to analyze changes in the soluble proteomic profile and cytolytic activity of *Millepora complanata* (“fire coral”) that underwent bleaching during the 2015–2016 El Niño-Southern Oscillation in the Mexican Caribbean Sea. Numerous investigations that have addressed the impact of elevated sea water temperature on reef forming cnidarians, from the first reports to the most recent ones, have evaluated the density of symbionts to assess the severity of bleaching [60,61,62]. In the case of the present study, we used the aforementioned criterion to determine the degree of bleaching of our collected samples. We found that bleached *M. complanata* specimens showed a decrease of 80% in the density of symbionts per square centimeter (Figure 1). The decline in symbiont density found in bleached *M. complanata* turned out to be similar to what has been reported in scleractinian species (symbiont density diminished by up to 50%–80%) [63,64,65,66,67,68]. It is worth mentioning that we conducted a parallel study on specimens of *M. alcicornis* collected in the same location and on the same dates as the *M. complanata* specimens. Interestingly, our study showed that bleached *M. alcicornis* specimens showed a decrease of 40% in the density of symbionts per square centimeter [48]. This suggests preliminarily that *M. alcicornis* is more thermotolerant than *M. complanata*. It would be very important to elucidate the mechanism underlying this thermotolerance.

It is well demonstrated that either in situ or experimental heat stress significantly affects the synthesis of proteins [40]. Accordingly, we found that bleached specimens of *M. complanata*, showed a 20% decrease in the total content of soluble proteins (Table 2). It is very likely that a lesser protein yield in the soluble proteome obtained from bleached specimens is related to the loss of protein components coming from zooxanthellae. The electrophoretic profiles of the soluble proteomes from both unbleached and bleached *M. complanata* holobiont were similar to those of the soluble proteomes from normal and bleached specimens of *M. alcicornis* [48]. Our results also agree with what has been previously observed in the protein profiles of other cnidarians, such as *H. magnipapillata* (class Hydrozoa), *A. elegantissima* (class Anthozoa), *C. fleckeri* (class Cubozoa), and *P. noctiluca* (class Scyphozoa) [69,70,71,72].

### 3.1. Levels of Proteins Implicated in Key Cellular Processes Were Modified in Bleached M. complanata

Seventy one proteins were found in both unbleached and bleached *M. complanata* holobiont-soluble proteomes, while the levels of 35 proteins were modified by bleaching. Mass spectrometric analysis of differential protein spots indicated that proteins whose levels were altered in bleached hydrocorals were involved in several cellular processes. Sixty one percent of these proteins showed amino acid sequence similarity to proteins that participate in important cell processes, such as: primary metabolism, DNA repair, cytoskeleton formation, signaling, stress response, redox homeostasis, and exocytosis (Figure 5). Bleached *M. complanata* specimens showed higher protein levels of alfa enolase, UV DNA endonuclease, HSP70, peroxiredoxin-6, and exocyst complex component 4 like protein. Whereas the protein levels of triosephosphate isomerase, DNA endonuclease repair XPF, actin, calmodulin, and hypothetical protein NEMVEDRAFT_v1g45829 were reduced. Worthy of mention is the fact that levels of four proteins, which displayed amino acid sequence similarity to cytolysins were augmented, including a phospholipase A2 (PLA2), an astacin-like metalloprotease toxin 5, and two pore forming toxins, echotoxin-2 and DELTA-actitoxin-Oor1b. In contrast, protein levels of an acidic calcium-independent phospholipase A2-like protein were reduced in bleached *M. complanata*.

Alpha enolase and triosephosphate isomerase were two enzymes, catalysing primary metabolic reactions, whose levels were altered in bleached hydrocorals. Alpha enolase is an enzyme that was previously identified in the transcriptome of *Hydra vulgaris* [73], which is phylogenetically close to *M. complanata*. This enzyme catalyzes the reversible conversion of 2-phosphoglycerate to phosphoenolpyruvate during both glycolysis and gluconeogenesis [74]. Higher level of alpha enolase in bleached *M. complanata* suggests increased activity in the glycolytic pathway. This result is in accordance with the response observed in *Acropora aspera* subjected to experimental bleaching, which showed a significant up-regulation of genes related to carbon metabolism (e.g., glycolysis, tricarboxylic acid cycle, and fatty acids synthesis [75]. Augmented alpha enolase level in bleached *M. complanata* might be a response to the diminished supply of energy due to the decrease in symbiont density, since glycolysis constitutes a major source of energy (in the form of ATP) and supplies the precursors for the synthesis of biomolecules such as lipids.

On the other hand, the level of triosephosphate isomerase was reduced in bleached *M. complanata*. This enzyme participates in glycolysis and gluconeogenesis, catalyzing the reversible synthesis of d-glyceraldehyde 3-phosphate from glycerone phosphate [76]. Our findings differ from the results obtained by Kenkel et al. (2013) who found an increase in the expression of genes encoding enzymes involved in gluconeogenesis in *Porites astroides* exposed to chronic heat stress [32]. Those authors proposed that the coral host balances its nutritional deficiency by converting their energetic reserves into carbohydrates. It has been demonstrated that under oxidative stress conditions, the expression of a subset of glycolytic proteins is repressed, while the expression of a few enzymes involved in the pentose phosphate pathway (PPP), which is directly connected to the glycolytic pathway, is induced [77]. Enzymes of the PPP are critical for preserving cytoplasmic NADPH concentration, which affords the redox power for antioxidant systems [78,79]. The observations above indicate that cells are capable of rerouting the carbohydrate flux from glycolysis to the PPP to counteract oxidative stress. Experiments carried out in *Saccharomyces cerevisiae* and *Caenorhabditis elegans* showed that reduction in triosephosphate isomerase expression or activity results in a redirection of the carbohydrate flux, which confers resistance against oxidative stress [80]. Considering the diminished levels of triosephosphate isomerase in bleached *M. complanata*, it is possible to hypothesize that thermal stress induces a decrease in triosephosphate isomerase expression in *M. complanata* as a mechanism to redirect the metabolic flux from glycolysis to the PPP in order to face oxidative stress. However, this hypothesis needs to be proven.

The levels of two DNA damage repair proteins were modified in bleached *M. complanata*. A protein which displays sequence homology with UVSE_BACCR, a component in a DNA repair pathway in *Bacillus cereus* [81], was up-regulated. Increased levels of this protein, which removes UV light-damaged nucleotides from DNA, could represent a response from *M. complanata* to repair the damage caused by high UV radiation and elevated seawater temperatures exposition. In contrast, lower levels of a protein that exhibited sequence homology with a DNA repair endonuclease XPF from the myxosporean *Thelohanellus kitauei* [82] were found in bleached *M. complanata*. Several studies have confirmed DNA damage in temperature-stressed corals, such as *Montastraea faveolata*, *Stylophora pistillata*, and *Acropora tenuis* [25,55,83]. Therefore, modified levels of UV DNA endonuclease and DNA endonuclease repair XPF supports that DNA damage occurs in *M. complanata* specimens that underwent bleaching.

Among the proteins whose levels were reduced in bleached hydrocorals was actin. Previous studies carried out on reef forming cnidarians have identified actin as a particularly sensitive protein to temperature stress [23,32,33,34,39,48]. In fact, actin genes have been proposed as a gene expression marker of heat stress that could be diagnostic of coral stress in the field [35]. The results obtained in the present study agree with what was observed in specimens of *Porites astreoides* [35] and *Stylophora pistillata* [36] subjected to heat stress, which demonstrated significant down-regulation of actin genes. In contrast, in the study we carried out on bleached *M. alcicornis* specimens, we found higher levels of actin [48], in a similar way to what was found in the scleractinian coral *Acropora palmata* [39]. The actin cytoskeleton is central in various cellular processes including cell motility, mitosis, intracellular transport, endocytosis, secretion, etc. [84,85]. Lower levels of actin in bleached *M. complanata* specimens may imply modifications in the intracellular transport, plasma membrane interactions, cell shape integrity and in the regulation of gene transcription of proteins that participate in cytoskeletal interactions.

Bleached *M. complanata* specimens also showed lower levels of calmodulin, which is a Ca^2+^ sensor protein, whose signaling is important in several cellular processes, such as cell cycle, apoptosis, intracellular transport, and calcium homeostasis [86]. This result is in agreement with what was found in the reef-building corals *Monstastraea faveolata* [23], *Acropora palmate* [28], and the symbiotic sea anemone, *Anemonia viridis* [87] exposed to experimental heat stress. Again, the results that we obtained in this study differ from what we found in bleached *M. alcicornis*, which exhibited higher abundance of calmodulin [48]. In the case of *M. complanata*, our results suggest that thermal stress provokes a disruption in cell calcium homeostasis. Undoubtedly, discrepancies found in the stress-responses related to the expression levels of actin and calmodulin between different reef forming cnidarians deserve further investigation.

Exposure of *M. complanata* to a thermally-induced bleaching event resulted in increased levels of heat shock protein 70 (HSP70). HSP70s are ubiquitous chaperones that facilitate correct protein folding and bind to partially denatured proteins to inhibit their aggregation. They are also able to renature denatured proteins conferring them repaired active states by an ATP-dependent way [88]. Since HSPs act as molecular chaperones preventing cellular damage under conditions of environmental stress, regulation of HSPs gene expression has been examined in scleractinian corals and *Symbiodinium* clades [24,37,88,89,90,91,92,93]. In general, thermal stress induces up-regulation of HSP70 gene expression in both *Symbiodinium* sp. and corals (*Acropora millepora*, *A. grandis*, *A. hyacinthus*, *Tubastrea cocchinea*, *Astrangia danae*, *Montastraea annularis*, *M. faveolata*, *Pocillopora damicornis*, and *Goniastrea aspera*) [24,37,88,89,90,91,92,93]. As expected, our results revealed a greater abundance of cytosolic HSP70 in *M complanata* exposed to heat stress. Considering that an improved thermotolerance in many marine organisms, including reef building corals, has been related to higher expression of stress-inducible members of the HSP70 family [90,94], it is very likely that up-regulation of HSP70 expression represents a heat-induced stress response of *M. complanata* to preserve protein structure and functions, and stimulate cellular repair processes to face global warming.

Similar to HSP70, peroxiredoxin-6 levels were also elevated in bleached *M. complanata*. This protein belongs to the family of peroxiredoxins, which neutralize oxidation products generated by reactive oxygen species (ROS) and therefore, protect cells from oxidative stress. Upregulation of this protein has been observed in *Acropora microphthalma* exposed to solar irradiance and heat stress [40]. Studies on the effect of heat stress on reef forming cnidarians have highlighted the important role that peroxiredoxins and other antioxidant enzymes, such as ascorbate peroxidase, superoxide dismutase, and catalase play to balance the oxidative damage generated by ROS during coral bleaching [95,96,97,98]. The observed increase in the levels of peroxiredoxin-6 indicates that *M. complanata* is dealing with ocean warming by activating its antioxidant mechanisms to prevent or revert damage provoked by ROS.

We also found that bleached *M. complanata* exhibited higher levels of an exocyst complex component 4 like protein in *M. complanata*. This protein has been proposed as a biomarker of coral heat stress [32,99], therefore our finding was to be expected. The exocytosis multiprotein complex has been related to the process of symbionts expulsion [100], since it offers spatial targeting of exocytotic vesicles to the membrane [101].

### 3.2. Proteins That Showed Amino Acid Sequence Similarity to Toxins Showed Different Levels in Bleached M. complanata 

Mass coral bleaching and mortality events that have occurred worldwide over the past three decades have caused great concern about the future of coral reef ecosystems [2,102]. Research on thermal tolerance of reef-forming corals indicates that some reef-forming cnidarians are able to deal with thermal stress, through specific adaptive processes, which include acclimatization, genetic adaptation, and symbiont shuffling, which may ameliorate the adverse consequences and mortality provoked by elevated sea water temperature [103,104,105,106]. Moreover, the ability to recover from a bleaching episode has been related to the energy reserves and heterotrophic feeding capacity of the cnidarian host [107,108,109]. *Symbiodinium* can provide more than 50% of their photosynthetic products to the cnidarian host [10,12,18,110,111,112]. However, after bleaching, recovering corals may heavily rely on alternate sources of fixed carbon, which is acquired via catabolism of energy reserves and/or by increased heterotrophy [113,114]. In fact, some evidence suggest that zooplankton provision may mitigate the negative impact of thermal stress [115]. 

*Millepora* species obtain nutrients from their autotrophic endosymbionts, however, they are also capable of capturing planktonic preys through heterotrophic feeding. Considering that autotropic input is significantly diminished during bleaching episodes [116], it is possible to hypothesize that under bleaching scenarios, *M. complanata* may increase the production of their chemical armament with the aim to balance the lack of energy from *Symbiodinium* algae.

As already mentioned above, the method we employed for obtaining the soluble proteomes from unbleached and bleached specimens of *M. complanata* involved osmotic shock in bidistilled water, which causes the discharge of the nematocysts content [48]. Interestingly, in the present study we found that bleached hydrocorals had increased levels of two proteins that showed amino acid sequence similarity to the pore forming toxins (PFTs), echotoxin-2 and DELTA-actitoxin-Oor1b, which were previously identified in “giant triton” *Monoplex parthenopeus* (phylum Mollusca) and the “Sea of Japan anemone” *Oulactis orientalis*, respectively [117,118].

DELTA-actitoxin-Oor1b belongs to the family of Actinoporins, which are the most abundant and best studied cnidarian PFTs [119,120]. These PFTs have been mainly identified in sea anemone venoms [120,121], although some actinoporin-like toxins have been found in other members of the class Anthozoa and in one species of the class Hydrozoa, *Hydra magnipapillata* [5,120,121,122]. Actinoporins are ~20 kDa proteins that spontaneously insert into sphingomyelin containing membranes [123]. In the case of actinoporin-like toxins from *Hydra*, they do not target sphingomyelin and display low sequence similarity (~30% identity) to actinoporins [124]. The actinoporin-like protein from *M. complanata*, which is predicted to have two α-helices, shares some functional features with three model actinoporins: DELTA-actitoxin-Aeq1a (Equinatoxin II; EqT II) and DELTA-actitoxin-Aeq1b (EqT V) from the “beadlet anemone” *Actinia equina* [125,126], and DELTA-actitoxin-Ucs1a (UcI) from the “Christmas anemone” *Urticina crassicornis* [127] (see Figure S1 of Supplementary material). The *M. complanata* actinoporin-like protein bears some conserved actinoporin binding site motifs and an aspartate that is present in the well-recognized actinoporin RDG-motif [128]. Noteworthy, the actinoporin-like protein we identified in *M. complanata* has an aromatic cluster motif that is similar to that of EqT II (W147, 151 and 152), which mediates the initial membrane attachment [129,130]. Most anemone actinoporins lack cysteine residues, however, *M. complanata* actinoporin-like protein owns one cysteine residue, which could include actinoporin-like toxins from *Stylophora pistillata* [122] and *Hydra magnipappilata* [131].

Augmented levels of the two pore forming like toxins from *M. complanata* correlated with increased hemolytic activity. Therefore, considering that PFTs are involved in processes such as feeding, digestion, defense, and spatial competition [120,121,128], it is very likely that upon loss of autotrophic input, *M. complanata* improves its heterotrophic capability as a strategy to counteract the loss of symbionts.

On the other hand, two proteins that exhibited homology to PLA2 displayed differential abundance in bleached hydrocorals. An acidic PLA2 PA4, previously reported in *Nemopilema nomurai* [132], showed elevated levels, whereas levels of an acidic calcium-independent PLA2-like, identified in the transcriptome of *Choristoneura fumiferana* [133], were diminished. At present, few cnidarian secreted phospholipases A2 have been isolated and structurally characterized [134,135,136,137,138] and it has been proposed that their functions comprise the capture and digestion of prey [139]. When assessing the PLA2 activity, we observed that the soluble proteome from bleached hydrocorals elicited a reduced enzymatic activity. This result is consistent with what we obtained in a previous study, in which we found that experimental thermal stress decreased the phospholipase A2 activity of an aqueous extract prepared from *M. complanata* [140]. Considering that the net PLA2 activity is the result of the sum of the effects induced by individual enzymes, our results suggest that the PLA2 activity induced by the soluble proteome of bleached *M. complanata* is mainly produced by enzymes, such as the acidic calcium-independent phospholipase A2 we detected, and other unidentified PLA2s, whose expression is very likely affected by thermal stress.

Another protein whose levels were raised in bleached hydrocorals showed homology (more than 30% amino acid sequence similarity) with astacin-like metalloprotease 5. This toxin is a zinc metalloprotease obtained from the spider *Loxosceles gaucho*, which provokes endothelial cells deadhesion and degradation of fibrinogen, fibronectin and gelatin [141]. The presence of metalloproteases has been described in several terrestrial animals venoms, such as those of snakes, spiders, centipedes, ticks [5,141,142,143], and also in soft-body cnidarians such as *Podocoryne carnea*, *Olindias sambaquiensis*, *Nematostella vectensis*, *Stomolophus meleagris*, *Nemopilema nomurai*, *Rhopilema esculenta*, *Cyanea nozakii*, *Aurelia aurita*, and *Chironex fleckeri* [5,144,145,146,147,148]. Metalloproteases from venomous animals appear to play an important role in envenomation, allowing the diffusion of toxic venom components by degradation of extracellular matrix. The over expression of this protein may be another indication that *M. complanata* is increasing the synthesis of toxins to improve its heterotrophic capacity in order to alleviate nutrient limitation derived from the impaired symbiotic relationship between hydrocorals and zooxanthellae. 

Interestingly, the results obtained in this study agree with what was recently found by Hoepner *et al.*, [149], who reported that the venom from the sea anemone *Entacmaea quadricolor*, exposed to long-term light-induced bleaching, preserve its hemolytic activity and lethality. These findings support the hypothesis that some cnidarians that have suffered bleaching are able to prey heterotrophically, giving them a better chance to resist the effects of thermal stress. 

## 4. Materials and Methods 

### 4.1. Sample Collection

*M. complanata* specimens were collected in the Parque Nacional Arrecife de Puerto Morelos, Quintana Roo, Mexico in November 2016 (Permission no. PFP/DGOPA-139/15). In order to avoid collecting identical clones, specimens (either bleached or unbleached) were collected from three colonies at depths of 4–10 m and at least 10 m apart. Sampled bleached and unbleached hydrocoral fragments (BMc and UMc, respectively) were frozen in liquid nitrogen and transported to the Laboratorio de Investigación Química y Farmacológica de Productos Naturales in the Universidad Autónoma de Querétaro, Mexico. This research project was approved by the Bioethics Committee of the Faculty of Chemistry of the Autonomous University of Querétaro (approved on 18 January 2017; approval code CBQ/17002).

### 4.2. Determination of the Degree of Bleaching

Fragments from unbleached and bleached *M. complanata* specimens were cut into squares of 1 cm^2^ (average weight = 0.9 g; average thickness = 0.5 cm). Subsequently, tissues were fixed in formalin buffer 10% for 3 days. Once the fixing time elapsed, samples were decalcified with HCl 5% during 5 days and the decalcifying solution was refreshed daily. Subsequently, tissues were homogenized in a Glas-Col homogenizer (IN, USA, Glas-Col) for 2 min at 70 rpm and centrifuged at 2400 rpm. The resulting pellet was resuspended in ethanol 70% and the number of symbionts was measured employing a Neubauer chamber. Experiments were performed in biological triplicates. Statistical differences between mean values were evaluated with a Student´s *t*-test using GraphPad Prism 6.0 (CA, USA, GraphPad Software).

### 4.3. Soluble Proteome Extraction from Bleached and Unbleached M. complanata 

Nematocyst discharge and soluble proteome extraction from unbleached and *bleached M. complanata* specimens was carried out employing osmotic shock. Hydrocoral fragments (~200 g) were immersed in distilled water (pH 7) at 4 °C and gently stirred for 24 h [8,150]. Afterward, the aqueous extracts obtained, containing the soluble proteomes, were centrifuged at 3000 rpm for 15 minutes at 4 °C, this procedure was repeated until solid insoluble detritus was no longer present. Subsequently, the supernatant was dehydrated using a lyophilizing system, and the resultant powder was stored at −70 °C. The concentrated lyophilized powders were dissolved in deionized water and protein concentration was measured using a bovine serum albumin standard curve, employing the Bradford´s method [151].

### 4.4. Electrophoresis SDS-PAGE

The soluble proteomes obtained from unbleached and bleached hydrocorals’ samples were precipitated with acetone and analyzed by one-dimensional SDS-polyacrylamide electrophoresis (SDS-PAGE), under denaturing and non-reducing conditions. A molecular weight standard (SDS-PAGE Molecular Weight Standards. Hercules, CA, USA, Bio-Rad) or 80 µg of protein were loaded in each well, samples were run in 12% polyacrylamide gels at 150 V during 1.5 h using electrophoresis buffer containing Tris, glycine, and SDS. Protein bands were visualized with Coomassie stain (Bio-Safe™ Coomassie Stain. Hercules, CA, USA, Bio-Rad).

### 4.5. First-Dimension Step, Isoelectric Focusing (IEF) and Two-Dimensional High-Resolution Gel Electrophoresis (2DE-PAGE)

For the first-dimension step, 750 µg of protein from the soluble proteomes of unbleached and bleached hydrocorals were cleaned using a clean-up Kit (Hercules, CA, USA, Bio-Rad) and solubilized in rehydration buffer containing 8 M Urea, 2% SDS, 0.375 Tris-HCl (pH 8.8), 20% glycerol and, 2% (w/v) DTT. Samples were loaded in an isoelectrofocusing system Bio-Rad PROTEAN^®^ i12™ (Hercules, CA, USA, Bio-Rad) and rehydrated overnight (100 V) on 11 cm immobilized pH gradient (IPG) strips ReadyStrip™ IPG pH 3–10 (CA, USA, Bio-Rad), for a total of 20,000 Vh. Experiments were performed in biological triplicates for both conditions. After the isoelectric focusing steps, the IPG strips were reduced using equilibration buffer I (urea 6M, 2% SDS, Tris–HCl 0.05 M, pH 8.8, 50% glycerol, and 2% (w/v) dithiothreitol (DTT)), and alkylated in equilibration buffer II (urea 6 M, 2% SDS, Tris–HCl 0.05 M, pH 8.8, 50% glycerol and 2.5% (w/v) Iodoacetamide). The second dimension separation was carried out using TGX Pre-Cast 18% SDS-polyacrylamide gels (CA, USA, Bio-Rad) at 150 V for 2 h at 4 °C. Gels containing both soluble proteomes were stained with BioSafe™ Coomassie blue G-250 dye (Hercules, CA, USA, Bio-Rad).

### 4.6. Image Analysis

Images of stained two-dimensional gels were captured in a Bio-Rad ChemiDoc™ MP (Hercules, CA, USA, Bio-Rad) imaging system at 600 dpi resolution assisted by the ImageLab™ (Hercules, CA, USA, Bio-Rad) software. Spot detection, matching, and fold changes were determined with the PD-Quest™ software (Hercules, CA, USA, Bio-Rad), version 8.0.1. Protein spots showing more than 2-fold significant difference in intensity between proteomes of BMc and UMc specimens were regarded as differentially expressed. These spots were selected and excised using an ExQuest™ spot cutter (Hercules, CA, USA, Bio-Rad) for further identification by MALDI-TOF/TOF MS. All experiments were performed in biological triplicates.

### 4.7. Protein in-Gel Digestion, MALDI-TOF/TOF Mass Spectrometry, and Data Analysis

Differentially expressed protein spots were excised from the two-dimensional electrophoresis gels and distained with ACN:NH_4_HCO_3_ 50 mM (50:50 v/v). The digestion of proteins was carried out during 15 h at 37 °C with mass spectrometry trypsin grade (Promega V528A). The resulting peptides were extracted with ACN:H_2_O:CH_2_O_2_ (50:45:5 v/v) and samples were desalted by chromatography using a C18 column ZipTipC18. Six technical replicates of each sample were loaded and co-crystallized in plates employing α-cyano-4-hydroxycinnamic as matrix and analyzed by means of a MALDI TOF/TOF 4800 analyzer (Concord, ON, CA, Applied Biosystems/ABsciex, 200 Hz, 355 nm Nd:YAG laser) coupled to the 4000 series Explorer software (version 3.5.3, Applied Biosystems Inc., Foster City, CA, USA). The operation mode was in positive ion and calibrated using a peptide mass standard kit for calibration (Applied Biosystems/ABsciex Concord, ON, CA). The laser was set to 2500–2800 for MS and 3500–3800 for MS/MS spectra acquisition. MS spectra were registered in positive ion reflector mode with 25 laser shots. Precursor ions were selected for fragmentation, which was carried out with a collision energy of 2 kV, employing air as collision gas at a pressure of 2 × 10–6 Torr with a total of 400 shots. Mass-charge spectra (*m*/*z*) were acquired within a molecular mass range of 800 to 4000 Da. The parental ion of Glu1-Fibrino-PeptideB, diluted in the matrix (1.3 pmol/µL/spot), was employed for internal calibration at *m*/*z* = 1570.690 Da. The 16 most intense ion signals per spot position having an *S*/*N* > 20 were selected for MS/MS acquisition. Following MALDI-TOF/TOF analysis, search and identification of partial sequences was performed using the ProteinPilot™ software (version 5.0, Applied Biosystems/ABsciex, Concord, ON, CA) and the Paragon searching algorithm (Applied Biosystems Inc., Foster City, CA, USA). The Basic Local Alignment Search Tool (BLASTp) (https://blast.ncbi.nlm.nih.gov/Blast.cgi) was used for identification of homolog sequences. The database was set to cnidaria (taxid: 6070) and non-redundant protein sequences (statistically significant scoring sequences with an E-value < 1 × 10^−6^ were retrieved). Similar sequences were aligned with the multiple sequence alignment program ClustalW2 (https://www.ebi.ac.uk/Tools/msa/clustalw2/).

### 4.8. Effect of Thermal Stress on the Cytolytic and PLA2 Activities of M. complanata Soluble Proteome

#### 4.8.1. Comparative Hemolytic Activity between the Soluble Proteomes from Unbleached and Bleached Specimens of M. complanata

In order to examine the difference between the hemolytic activity of the soluble proteomes from unbleached and bleached hydrocorals, a hemolytic test was performed according to a method previously described [150]. The hemolytic assay is currently used to evidence the presence of cytolysins. Different protein concentrations (µg/mL) of three biological replicates were mixed with Alsever’s solution (120 mM d-glucose, 30 mM sodium citrate, 7 mM NaCl, and 2 mM citric acid, pH 7.4) and 50 µL of 1% erythrocytes suspension, this mixture was incubated at 37 °C for 30 min. Samples were centrifuged at 1500 rpm for 4 min at 4 °C. The absorbance at 415 nm of the supernatant fluid, containing the hemoglobin released from lysed erythrocytes, was measured in a Benchmark Plus microplate spectrophotometer (Hercules, CA, USA, Bio-Rad). Each experiment was normalized with respect to complete hemolysis, which was measured by diluting the erythrocyte sample in deionized water instead of Alsever’s buffer. One hemolytic unit (HU_50_) was defined as the amount of protein sample required to cause 50% hemolysis. The hemolytic activity was plotted in GraphPad Prism 6.0 (Hercules, CA, USA, GraphPad Software). Statistical difference between mean values from normal and bleached samples was evaluated by a Student´s *t*-test.

#### 4.8.2. Comparative PLA2 Activity between the Soluble Proteomes from Unbleached and Bleached Specimens of *M. complanata*

The PLA2 activity of the soluble proteomes from three specimens of each unbleached and bleached *M. complanata* was measured employing a secretory PLA2 colorimetric assay kit (Ann Arbor, MI, USA, Cayman Chemical) according to the manufacturer’s protocol. The PLA2 activity test is employed to measuring the activity of this toxic enzyme from venoms. Briefly, the assay uses diheptanoyl phosphatidylcholine as a substrate, and the PLA2 activity is detected by the generation of free thiols from hydrolysis of the sn-2 thioester bonds of the substrate. Changes in coloring were measured with a Benchmark Plus microplate spectrophotometer at 414 nm (Hercules, CA, USA, Bio-Rad). The PLA2 activity was expressed as micromoles of hydrolyzed substrate per minute per milligram of protein. Curve of PLA2 activity was plotted with GraphPad Prism 6.0 (San Diego, CA, USA, GraphPad Software). Statistical difference between mean values from normal and bleached samples was evaluated by a Student´s *t*-test.

## 5. Conclusions

This study presented evidence demonstrating that the El Niño–Southern Oscillation 2015–2016 induced a significant decrease in symbiont density in some colonies of *M. complanata* that inhabit the Mexican Caribbean, indicating that these hydrocorals underwent a severe bleaching. The levels of proteins involved in key cellular processes, such as glycolysis, DNA repair, stress response, calcium homeostasis, exocytosis, and cytoskeleton organization were significantly modified in bleached hydrocorals. Four of the proteins, whose levels were augmented, exhibited amino acid sequence similarity to pore-forming toxins, a phospholipase A2, and a metalloprotease. Accordingly, the hemolytic effect of the soluble proteome of bleached hydrocorals was significantly higher. These results allowed us to infer that bleached *M. complanata* is capable of increasing its toxins production in order to balance the negative impact of elevated temperature on its autotrophic nutrient input. This may represent a resilience mechanism by which hydrocorals face thermal stress.

## Figures and Tables

**Table 1:** Identification of differentially abundant proteins associated with bleaching in *M. complanata* holobiont by MALDI-TOF/TOF, ProteinPilot search engine, and BLASTp analysis.

Spot	Protein	Accession Number	MW/pI ^a^	P ^b^	Log2-fold Change
**Toxins**					
2001	Acidic PLA2 PA4	A0A1D8GZE6_9CNID	16.0/4.7	↑	4.4
2201	Echotoxin-2	ACTP2_MONPT	39.7/4.6	↑	3.1
3103	DELTA-actitoxin-Oor1b	ACTPG_OULOR	22.805.1	↑	5.6
7101	PREDICTED: Astacin-like metalloprotease toxin 5	XP_002162822.1	33.1/7.0	↑	2.2
5001	Acidic calcium-independent phospholipase A2-like protein	Q307P2_CHOFU	19.1/5.8	↓	0.3
**Primary metabolism**					
5406	Alpha enolase	T2MHB9_HYDVU	53.2/6.3	↑	2.8
7104	PREDICTED: triosephosphate isomerase	XP_0021676111	27.6/6.7	↓	0.4
**DNA repair**					
3104	UV DNA endonuclease	UVSE_BACCR	28.5/5.1	↑	3.0
6002	DNA endonuclease repair XPF	A0A0C2MR15_THEKT	12.5/6.7	↓	0.04
**Cytoskeleton Component**					
5408	Actin	ACT_HYDVU	47.0/6.1	↓	0.3
**Signaling protein**					
1101	Calmodulin	T2MET0_HYDVU	22.7/4.7	↓	0.4
**Stress response**					
5504	HSP70	HSP70_HYDVU	70.0/5.9	↑	2.6
**Homeostasis redox**					
6103	Peroxiredoxin-6	T2MGB9_HYDVU	22.8/6.2	↑	4.7
**Exocytosis protein**					
7103	PREDICTED: exocyst complex component 4-like protein	XP_004208568.1	33.2/7.5	↑	2.2
**Unknown**					
6206	Hypothetical protein NEMVEDRAFT_v1g45829	A7TDG2_NEMVE	34.7/6.6	↓	0.3

^a^ Molecular weight/Isoelectric point; ^b^ Protein levels. ↑ indicates higher protein levels comparing to those of unbleached specimens. ↓ indicates lower protein levels comparing to those of unbleached hydrocorals

**Table 2:** Cytolytic activity of the soluble proteomes of UMc and BMc.

Extracts	Soluble Protein Content ^a^	Hemolytic Activity ^b^	PLA2 Activity ^c^
UMc	31.04 ± 1.30	2.07 ± 0.35	124.70 ± 1.98
BMc	22.02 ± 0.70	0.96 ± 0.08 *	29.13 ± 1.26 *

^a^ Soluble protein content expressed in μg protein/mg lyophilized; ^b^ Hemolytic unit (HU_50_) expressed in μg protein/mL; ^c^ PLA2 Activity expressed in μmol min^−1^mg^−1^); * *p* < 0.05.

## Supplementary Materials

The following are available online at https://www.mdpi.com/1660-3397/17/7/393/s1, Supplementary Figure 1: Alignments of the actinoporin-like protein from *M.complanata* (Spot 3103 SEC-4) and three model actinoporins.
