# Supplementary material for: Comparative Analysis of the Soluble Proteome and the Cytolytic Activity of Unbleached and Bleached Millepora complanata (“Fire Coral”) from the Mexican Caribbean
Source: Mar Drugs. 2019 Jul 3;17(7):393. doi: 10.3390/md17070393 (PMC6669453; doi:10.3390/md17070393)
Supplement: Supplementary file 1 [file marinedrugs-17-00393-s001.pdf]

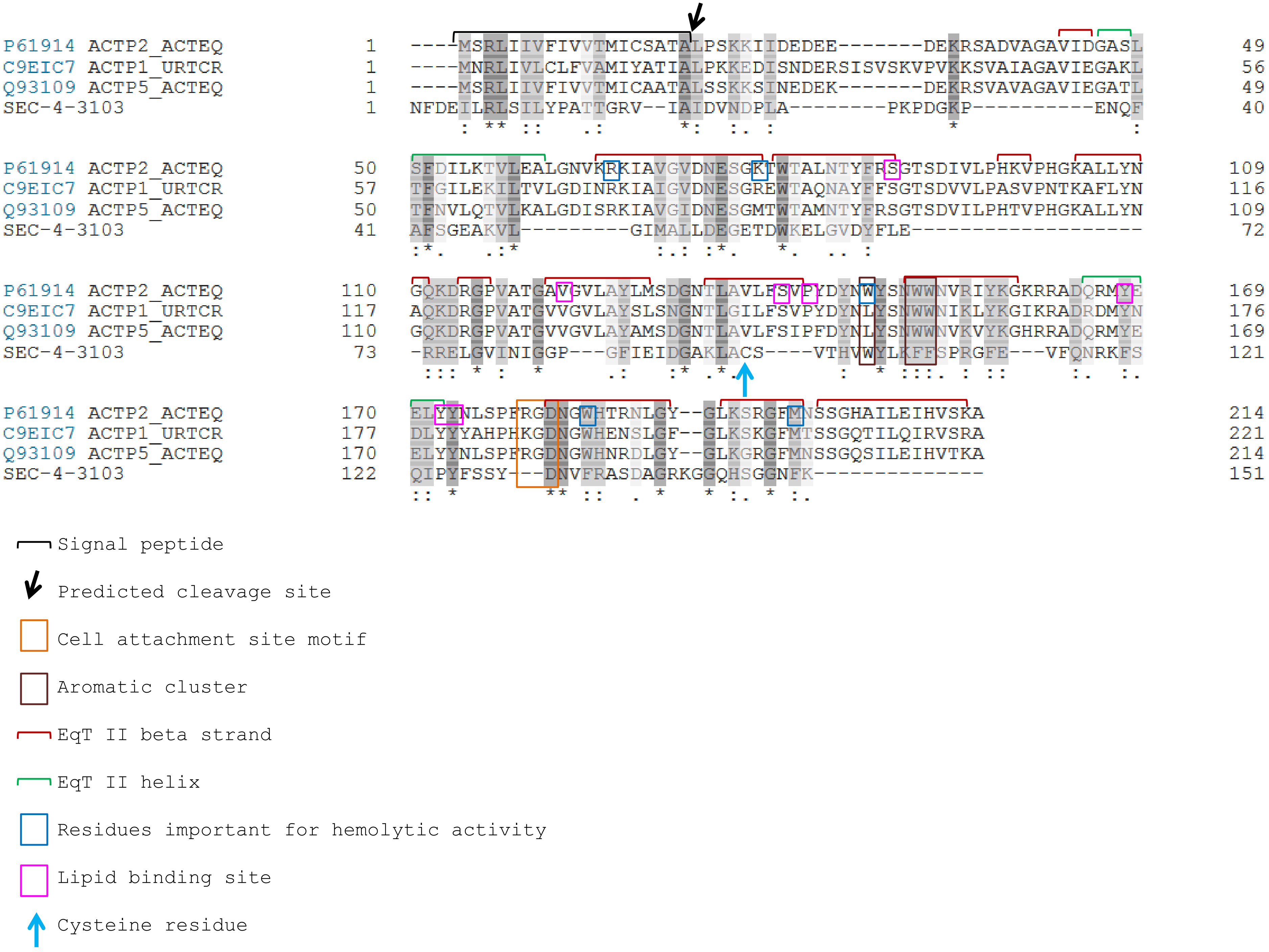

Supplementary Figure 1. Alignments of the actinoporin-like protein from *M.complanata* (Spot 3103 SEC-4) and three model actinoporins. The accession numbers of the sequences used are as follows: DELTA-actitoxin-Aeq1a (Equinatoxin II from *Actinia equina*): P61914.; DELTA-actitoxin-Ucs1a (*Urticina crassicornis*): C9EIC7.; DELTA-actitoxin-Aeq1b (*Actinia equina*): Q93109. Sequences were aligned with ClustalW (default parameters). Identic (*), conserved(:) and, semi-conserved (.) amino acid residues are displayed.
